# Effects of Umami Taste on Visual Food Cue Processing: An ERP Study with Source Localization

**DOI:** 10.3390/foods14142409

**Published:** 2025-07-08

**Authors:** Anne Schienle, Florian Osmani, Emilia Iannilli

**Affiliations:** Institute of Psychology, University of Graz, 8010 Graz, Austria

**Keywords:** umami, monosodium glutamate, visual food cues, event-related potentials, late positivity, source localization, desire to eat, fusiform gyrus

## Abstract

Research suggests that monosodium glutamate (MSG), known for its association with umami taste, may influence appetite. This event-related potential (ERP) study with source localization examined the effects of umami taste on visual food cue reactivity. A total of 88 females (mean age: 26 years) were randomly assigned to one of two groups: one tasted an MSG solution, while the other tasted water, before viewing images from four food categories with varying umami concentrations (meat dishes, vegetables, fruits, sweets). The participants rated their desire to eat the depicted foods, and ERP late positivity (P300; late positive potential: LPP) was analyzed. The results showed that MSG did not influence reported appetite. However, the MSG group exhibited reduced parietal P300/LPP amplitudes across all food categories. These group differences in food cue processing could be traced back to sources in the left occipital gyrus and fusiform gyrus indicating an alteration in motivated attention due to umami taste.

## 1. Introduction

Glutamate is a naturally occurring amino acid found in protein-rich foods such as beef and seafood [1]. It is also present in high concentrations in certain vegetables, including tomatoes and mushrooms. Additionally, glutamate is commonly added to processed foods such as soups, salty snacks, fast food (e.g., chips, burgers), and condiments (e.g., ketchup, mayonnaise) in the form of monosodium glutamate (MSG). The food industry uses MSG to enhance flavor and increase consumer desire for these products [2].

Glutamate/MSG is associated with umami taste, which is one of the five basic tastes besides sweet, bitter, salty, and sour [3,4]. Umami (from Japanese) translates to ‘pleasant savory taste’. Research has shown that MSG as a food additive leads to increased perceived food palatability [5], along with more positive ratings for the flavor and acceptability of food [6]. In this way, MSG possesses the characteristics of an appetite enhancer. More specifically, an umami sensation indicates the existence of proteins in meals, leading to an increased appetite for proteins [2]. However, recent research has also documented satiety-increasing effects of MSG. For example, Masic and Yeomans [7] added MSG to a low-energy meal. This resulted in a biphasic effect: MSG enhanced appetite during eating but also satiety after the meal. However, more research is needed to further distinguish the satiating and appetite-enhancing effects of umami [8].

Other basic tastes have also been linked to food cue reactivity, including appetite modulation. For example, bitterness—often signaling potential food spoilage, especially at high concentrations—reduces the reported desire to eat that food [9]. In an ERP study by Schwab et al. [10], which used the same design as the present investigation, healthy-weight participants viewed food images after rinsing their mouths with either a bitter fluid (wormwood) or water. The bitter taste reduced the amplitude of the P300 across a fronto-central cluster.

The P300, a positive deflection in the electroencephalogram occurring approximately 300 ms after stimulus onset, reflects stimulus salience and attentional processes [11]. Research on visual food cue processing has consistently shown that food images elicit increased (parietal) amplitudes of the P300 and the subsequent late positive potential (LPP) compared to non-food images. Additionally, high-calorie foods generate higher P300/LPP amplitudes than low-calorie foods [12,13]. P300/LPP amplitudes have also been positively correlated with appetite and food intake [14,15].

Sources of parietal late positivity have been localized within the visual association cortex [16]. A meta-analysis by Huerta et al. [17] comparing neural responses to food versus non-food images found consistent activation in the right fusiform gyrus.

The present study examined the effect of umami taste on visual food cue processing. Healthy female participants viewed images of foods categorized as sweets, meat dishes, fruits, and vegetables. Higher concentrations of glutamate are found in meat dishes (e.g., beef) and certain vegetables (e.g., tomatoes), whereas sweet dishes contain considerably lower levels [1]. Before viewing the images, the participants tasted (and ingested) either an MSG solution or water (control group). This study analyzed whether umami would influence the desire to eat the depicted foods and ERP late positivity (P300, LPP) in response to the food images. Additionally, an exploratory source localization analysis was performed.

## 2. Materials and Methods

### 2.1. Participants

Ninety females were recruited using printed flyers, social media platforms, and email distribution lists at the university. The sample was restricted to females because of sex differences in reported food preferences and umami pleasantness [18]. This approach was chosen to reduce sex-related biological and behavioral variability in the data, allowing for the detection of smaller effects with greater statistical power. Data from two participants had to be excluded due to technical problems. The participants were randomly assigned to one of the two taste conditions: water (*n* = 44) or MSG (*n* = 44). The groups did not differ in their mean age (t(86) = 0.60, *p* = 0.28) or body mass index (t(86) = 1.98, *p* = 0.051; Table 1). The majority of the participants were university students (72%) and described their dietary style as omnivorous with low meat consumption.

### 2.2. Stimuli

*Images:* A total of 120 different food pictures were selected from Food-Pics [19] representing four categories: sweets (e.g., cakes, ice cream), meat dishes (e.g., burgers, steaks), vegetables (e.g., tomatoes, mushrooms), and fruits (e.g., apple, watermelon). Each picture category consisted of 30 pictures. The categories sweets and meat, as well as vegetables and fruits, were matched for calorie contents (the number of calories per 100 g). The pictures were shown in random order for 1500 ms each and were preceded by a fixation cross (500–1000 ms). Five pictures per category (random selection before the experiment) were evaluated by the participants during the EEG experiment according to ‘liking’ (How much do you like this food item in general?) and ‘wanting’ (How much would you like to eat this food item right now?) on Likert scales (1 = no at all; 100 = extremely).

*Fluids*: The participants tasted and ingested a 10 mL MSG solution (L-glutamic acid monosodium salt monohydrate, C5H8NNaO_4_-H_2_O, from Sigma-Aldrich Chemistry, Steinheim am Albuch, Germany) or 10 mL water. MSG was diluted in water to a concentration of 241 mM (supra-threshold) based on previous research [18]. The fluids were rated according to pleasantness (How pleasant is this taste for you?), perceived intensity (How intense is this taste?), and familiarity (How familiar are you with this taste?) on 100-point Likert scales (0 = low; 100 = high).

### 2.3. Experimental Procedure

This study was preregistered at the German Register for Clinical Studies (DRKS00031564, 25 April 2023), followed the Declaration of Helsinki, and was approved by the local ethics committee (GZ.39/83/63 ex 2022/23). All participants provided informed consent.

**Figure 1:**
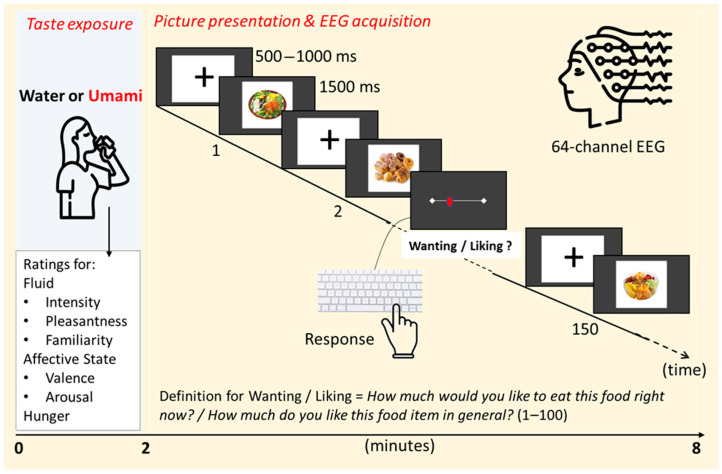
Experimental design.

The participants were asked to refrain from eating for four hours before coming to the EEG laboratory. At the beginning and the end of the experiment, the participants rated their levels of hunger and evaluated their affective state according to positive valence and arousal (0 = low; 100 = high). The pictures were presented by means of PsychoPy2^®^ software, [20]. The picture presentation trigger was registered synchronously to the EEG signal. After the experiment, a taste discrimination task using MSG and NaCl solutions was conducted to identify and exclude potential umami non-tasters. However, no parti-cipants met the exclusion criteria. The design of the experiment is illustrated in Figure 1.

A power analysis for two groups and four picture categories (power = 0.80, α = 0.05, a small effect size of f = 0.12) indicated a required total sample size of n = 90.

### 2.4. EEG Recordings and Data Analysis

The EEG was recorded with a BrainVision actiCHamp amplifier, 64-channel actiCAP active electrodes, and BrainVision Recorder software (Brain Products GmbH, Gilching, Munich, Germany). The acquisition sampling rate was set at 2500 Hz with a passband filter of 0.016–1000 Hz. The data analysis was carried out by means of Cartool software v4.11 [21], following current best-practice steps to estimate the distribution of neuronal sources derived from multichannel EEG recordings [22]. The pre-processing steps included data resampling to 250 Hz, epoching (1500 ms interval with 200 ms peristimulus also used as a baseline), filtering (low-pass 30 Hz, high-pass 0.1 Hz, and notch 50 Hz), baseline correction, re-referencing to the linked mastoid, and manually rejecting artifacts due to eye movements after visual inspection. On average, eight trials per participant were excluded due to artifacts. Participants with fewer than eight artifact-free trials were excluded from further analyses. For each participant, a grand average (GA) was computed for each condition. The single-subject GA was entered into the group grand mean (GM) for a total of 8 GMs: 2 groups (water, umami) × 4 food categories (sweets, meat, vegetable, fruits).

Following the analysis plan by Schwab et al. [10], mean event-related potentials (ERPs) were extracted for the time windows 250–400 ms (P300) and 400–1000 ms (LPP). Mean activation was aggregated across frontal (F3, Fz, F4), central (C3, Cz, C4), and parietal (P3, Pz, P4) clusters separately for the P300 and LPP.

### 2.5. Source Localization

The localization of the EEG sources was achieved by co-registering the EEG electrodes placed on the skin with an MNI template (Montreal Neurological Institute) [23] with predefined translation parameters to match the two spaces. The coordinates of the localized areas were converted into Talairach space, which is the reference framework utilized by the brain atlas [24]. As a head model, we used a local spherical model with anatomical constraints (LSMAC) with space solution constrained to the gray matter for a total of 6000 solution points. A local auto-regressive average (LAURA) [25] algorithm was applied to estimate the inverse solutions. For the EEG source analysis, we utilized an artifact-free dataset from 86 participants (water: *n* = 44, MSG: *n* = 42).

### 2.6. Statistical Analyses of the Rating Data

A mixed analysis of variance (ANOVA) was performed to compare participants’ ratings for their current hunger level and affective state (valence, arousal) between groups (MSG, water) before and after the experiment (within-subject factor: time).

T-tests were computed to compare the taste ratings for the fluids (valence, intensity, familiarity) between groups.

Mixed ANOVAs were performed to compare groups and food categories (sweets, meat, vegetable, fruits) concerning self-reports (food wanting and liking) and P300/LPP amplitudes across the frontal, central, and parietal clusters.

## 3. Results

### 3.1. Ratings

The descriptive statistics for all rating data are displayed in Table 1.

*Hunger level:* The effect for time was significant (F(1,85) = 22.68, *p* < 0.001, ηp^2^ = 0.21). The reported hunger level was higher after the experiment (M = 58.31; SD = 30.89) than before the experiment (M = 48.77, SD = 26.38). The effects for group (F(1,85) = 1.02, *p* = 0.315) and the interaction time × group (F(1,85) = 0.07, *p* = 0.796) were not significant.

*Valence (affective state):* The effect for time was significant (F(1,86) = 10.17, *p* = 0.002, ηp^2^ = 0.11). The reported valence was higher before the experiment (M = 76.94; SD = 17.59) than after the experiment (M = 71.25, SD = 19.58). The effects for group (F(1,86) = 0.03, *p* = 0.860) and the interaction time × group (F(1,86) = 0.07, *p* = 0.789) were not significant.

*Arousal (affective state)*: The effect for time was significant (F(1,85) = 17.72, *p* < 0.001, ηp^2^ = 0.17). The reported arousal was higher before the experiment (M = 28.68; SD = 23.31) than after the experiment (M = 20.54, SD = 20.13). The effects for group (F(1,85) = 0.65, *p* = 0.423) and the interaction time × group (F(1,85) = 0.09, *p* = 0.765) were not significant.

*Fluid ratings:* The MSG group rated the fluid as less pleasant (t(86) = 10.68, *p* < 0.001), less familiar (t(86) = 11.64, *p* < 0.001), and more intense (t(86) = 9.88, *p* < 0.001) than the water group (Table 1).

*Picture ratings (food liking):* The effect for food category was significant (F(3,258) = 46.32, *p* < 0.001, ηp^2^ = 0.35). Fruits received higher liking ratings than all other food categories (*p* < 0.001). Meat received lower liking ratings than all other categories (*p* < 0.001). The effects for group (F(1,86) = 0.05, *p* = 0.818) and the interaction group x food category (F(3,258) = 0.66, *p* = 0.575) were not significant.

*Picture ratings (food wanting):* The effect for food category was significant (F(3,258) = 49.46, *p* < 0.001, ηp^2^ = 0.37). Fruits received higher ratings for wanting than all other food categories (*p* < 0.001). Meat received lower ratings for wanting than all other categories (*p* < 0.001). The effects for group (F(1,86) = 0.07, *p* = 0.785) and the interaction group × food category (F(3,258) = 0.77, *p* = 0.514) were not significant.

### 3.2. Event-Related Potentials

*Frontal P300:* The effect for food category reached statistical significance (F(3,258) = 4.41, *p* = 0.005, ηp^2^ = 0.05). The effects for group (F(1,86) = 0.67, *p* = 0.416) and the interaction group × category (F(3,258) = 0.90, *p* = 0.440) were not significant. Fruits (M = −0.44, SD = 4.05) were accompanied by higher amplitudes (less negative values) compared to meat (M = −1.24, SD = 4.13) and vegetables (M = −1.68, SD = 3.37; *p* < 0.008). Fruits and sweets (M = −1.12, SD = 3.84) did not differ from each other (*p* = 0.067).

*Central P300:* The effect for food category reached statistical significance (F(3,255) = 3.00, *p* = 0.031, ηp^2^ = 0.03). The effects for group (F(1,85) = 0.90, *p* = 0.345) and the interaction group × category (F(3,255) = 0.62, *p* = 0.601) were not significant. Fruits (M = 0.38, SD = 1.65) were accompanied by higher amplitudes compared to sweets (M = 0.12, SD = 1.65, *p* = 0.007), meat (M = 0.19, SD = 1.83, *p* = 0.041), and vegetables (M = 0.09, SD = 1.62, *p* = 0.007).

**Figure 2:**
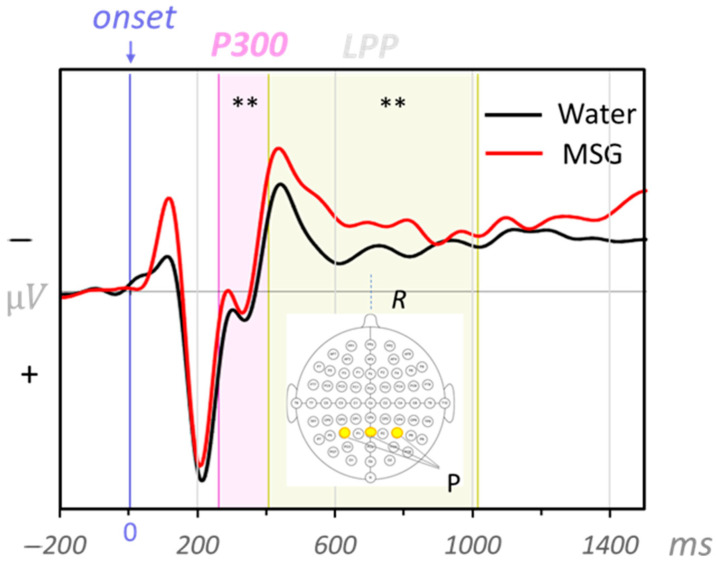
ERP grand averages across the parietal cluster for P300/LPP components for the groups (MSG: monosodium glutamate, water) across all food images. P: parietal cluster. Significant group effects are highlighted with ** (*p* < 0.01).

*Parietal P300:* The effect for group (F (1,85) = 13.79, *p* < 0.001, ηp^2^ = 0.14) was statistically significant. The effects for food category (F(3,255) = 1.75, *p* = 0.158, ηp^2^ = 0.02) and the interaction group × category were not significant (F(3,255) = 0.17, *p* = 0.915, ηp^2^ = 0.002). The MSG group showed lower amplitudes (M = 0.89, SD = 1.97) compared to the water group (M = 2.56, SD = 2.21; see Figure 2).

*Frontal LPP*: The effect for food category was statistically significant (F(3,255) = 11.71, *p* < 0.001, ηp^2^ = 0.12). The effects for group (F(1,86) = 0.02, *p* = 0.893) and the interaction group × category (F(3,258) = 0.79, *p* = 0.501) were not significant. Fruits (M = 2.52, SD = 3.35) were accompanied by higher amplitudes compared to sweets (M = 1.02, SD = 3.22, *p* < 0.001), meat (M = 0.65, SD = 3.64, *p* < 0.001), and vegetables (M = 0.35, SD = 3.31, *p* < 0.001). Fruits and sweets did not differ from each other (*p* = 0.069)

*Central LPP:* The effect for food category was statistically significant (F(3,255) = 12.99, *p* < 0.001, ηp^2^ = 0.13). The effects for group (F(1,85) < 0.01, *p* = 0.965) and the interaction group × category (F(3,255) = 0.32, *p* = 0.812) were not significant. Fruits (M = 1.12, SD = 1.25) were accompanied by higher amplitudes compared to sweets (M = 0.62, SD = 1.02, *p* < 0.001), meat (M = 0.54, SD = 1.24, *p* < 0.001), and vegetables (M = 0.32, SD = 0.97, *p* < 0.001). Sweets were accompanied by higher amplitudes compared to vegetables (*p* = 0.006). Meat and vegetables did not differ from each other (*p* = 0.089)

*Parietal LPP*: The effects for group (F (1,85) = 16.61, *p* < 0.001, ηp^2^ = 0.16) and food category (F(3,255) = 3.86, *p* = 0.010, ηp^2^ = 0.04) were statistically significant. The interaction group x category was not significant (F(3,255) = 2.41, *p* = 0.068, ηp^2^ = 0.03). Fruits (M = 0.83, SD = 2.77) were associated with higher amplitudes compared to meat (M = 0.07, SD = 3.39, *p* = 0.006) and vegetables (M = 0.03, SD = 2.84, *p* = 0.010). Fruit and sweets (M = 0.48, SD = 3.18) did not differ from each other (*p* = 0.172). The MSG group had lower amplitudes (M = −0.71, SD = 1.75) compared to the water group (M = 1.39, SD = 2.91; see Figure 2).

### 3.3. Source Localization and Estimated Current Source Density (eCSD)

**Figure 3:**
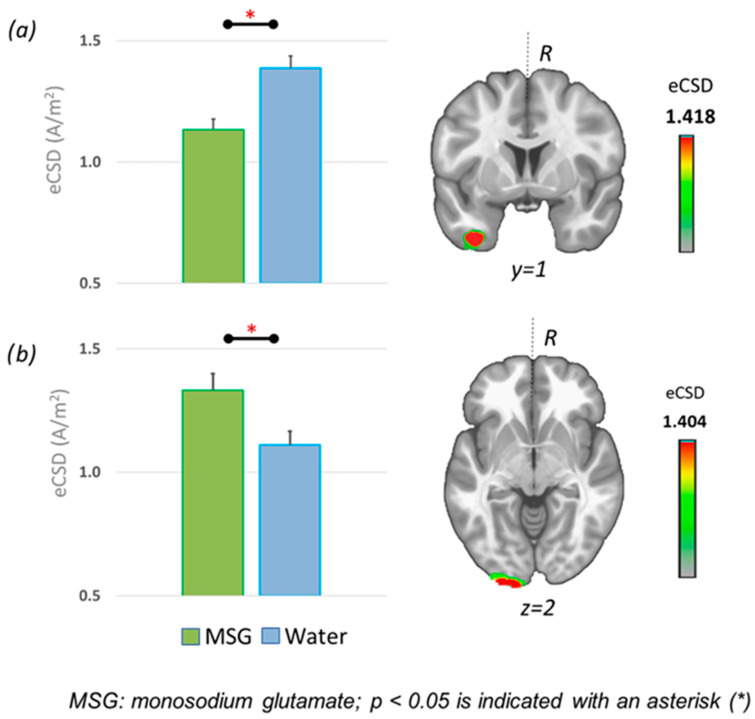
A comparison of estimated current source density (eCSD; means, standard errors) between the two groups (MSG, water) for the combined four food categories in the (**a**) left fusiform gyrus and (**b**) left occipital gyrus in the LPP window.

Based on the ERP findings, we extracted each participant’s estimated current source density (eCSD) for the combined four food categories from the identified solution point corresponding to the sources in the P300 and LPP windows. The identified sources included the right and left fusiform gyrus (P300, LPP), the left occipital gyrus, and the right frontopolar gyrus (both LPP; see Table 2). Subsequently, the eCSDs were compared between the groups (water, MSG) via t-tests (Figure 3).

*P300 window*: No significant group differences were detected in the right fusiform (t(84) = −1.292, *p* = 0.100).

*LPP window*: The groups differed in their eCDS in the left fusiform gyrus (t(84) = 2.806, *p* = 0.003) and left occipital gyrus (BA17, primary visual cortex) (t(84) = −2.272, *p* = 0.013). No significant differences were detected between groups in the right frontopolar gyrus (t(84) = 0.256, *p* = 0.399).

## 4. Discussion

To the best of our knowledge, this ERP study is the first to investigate the influence of umami taste (induced via monosodium glutamate, MSG) on the processing of visual food cues. On average, the MSG solution was perceived as an unpleasant gustatory stimulus. This is in line with previous studies [18,26] in which the participants rated an MSG solution (which had the same intensity as in the present investigation) as mildly unpleasant [18]. Similar ratings indicating a negative hedonic value of an MSG solution have also been reported for Asian individuals. In a study by Zhi et al. [27], participants tasted different solutions (e.g., MSG, sugar, NaCl) while their facial expressions were recorded. Umami was associated with expressions of disgust and fear, particularly in high concentrations.

In the present investigation, umami did not influence the ratings for the food images (wanting, liking). The two groups (MSG and water) did not differ in their reported desire to eat the depicted salty foods high in glutamate (e.g., beef, vegetables) or foods low in glutamate (e.g., sweets and fruits). This finding contrasts with those of previous studies, which, however, used larger amounts of MSG added to meals [7]. Independent of the group assignment, the participants experienced an increase in hunger over the course of the experiment. This is a well-known phenomenon: reported hunger/appetite increases due to repeated exposure to visual food cues [28].

In contrast to the unaffected self-reports, MSG reduced late positivity in the EEG. The MSG group was characterized by lower amplitudes of the P300 and the LPP for all food categories across the parietal cluster. Thus, there were no differential effects of umami on the selected food categories with different MSG concentrations. It has been previously suggested that umami signals the presence of amino acids, encouraging the consumption of specific protein-rich foods. However, many foods that naturally contain high levels of glutamate are not generally high in protein, such as peas, corn, and tomatoes [4,29]. Similarly, high-protein foods like pork and chicken do not contain significant amounts of glutamate [30]. Thus, the identification of food high in glutamate is not easy based on visual inspection alone or knowledge about its macronutrients.

The results of the source localization analysis indicated group differences in eCSD concerning the left occipital gyrus (BA17) and the left fusiform gyrus in the LPP window. The MSG group displayed higher activity in BA17, which corresponds to the primary visual cortex (V1). It has been shown that when attention is focused on an object, V1 neurons responding to that object fire more, making it more visually dominant. This enhances object recognition [31]. Thus, umami enhanced perceptual processing of the food cues.

Surprisingly, activity in the left fusiform gyrus (FFG) was lower in the MSG group than in the water group. Neuroimaging research on the visual processing of food cues has consistently shown that food images activate the FFG (for a review and meta-analysis, see [17,32]). Jain et al. [33] identified a specific subdivision of the FFG that is selectively responsive to food images, located in the left hemisphere, aligning with our findings. Moreover, FFG activation reflects motivated attention. For example, when we are hungry or craving something, the brain prioritizes food images, leading to stronger FFG activation [34]. In contrast, the MSG-associated reduction in FFG activity as observed in the present study suggests a decreased motivational value of food stimuli [11]. This finding is unexpected, given previous research identifying MSG as an appetite enhancer [2]. However, in our study, MSG was administered in its pure form rather than as a food additive. This method of administration was perceived as unpleasant [18,27] and induced EEG late positivity changes similar to those observed in a previous bitter-taste study [10].

We need to acknowledge the following limitations of our study. First, our sample consisted of highly educated females from Austria; therefore, the results cannot be generalized to other segments of the general population. Future studies should include male participants using the same experimental design to allow for direct comparison with the current findings.

Second, several participants reported reduced meat intake in their general diet. Consistent with this dietary style, fruits received higher ratings for liking and wanting than meat and were accompanied by higher P300/LPP amplitudes than the other food categories. Therefore, a future investigation should include participants who indicate a greater liking of meat dishes.

Third, ratings for wanting and liking of food are susceptible to social desirability effects. Of note, the participants reported a preference for healthy, low-calorie foods. However, it remains uncertain whether these stated preferences would translate into actual choices in a behavioral context, such as a food tasting task. Future studies should address this aspect, for example, by employing a bogus taste test paradigm to assess real food selection behavior. Additionally, implicit measures may be valuable for capturing affective and attitudinal responses toward food stimuli that participants may be unwilling or unable to report consciously. Such methodologies are particularly effective in circumventing the confounding effects of social desirability. Moreover, other behavioral tests could be introduced. For example, Xiao et al. [35] used a taste–visual Stroop task—requiring participants to rapidly judge the flavor of the food depicted in an image by pressing the appropriate response key—to investigate cross-modal processing. Participants were presented with either congruent stimulus pairs (e.g., images depicting sour foods paired with a sour taste) or incongruent pairs (e.g., images depicting sour foods paired with a sweet taste). Stroop interference was reflected in a negative difference component observed between 430 and 620 ms.

Finally, given the participants’ reported limited familiarity with umami taste, this study highlights the necessity of conducting cross-cultural investigations.

## 5. Conclusions

Although MSG consumption did not significantly alter self-reported appetite (food wanting), the findings suggest that umami taste modulates the brain’s attentional response to food cues. Specifically, the reduced P300/LPP amplitudes and associated activity in the occipital and fusiform gyri indicate that MSG can influence motivated attention toward food images. These findings highlight the potential of umami taste to affect food-related processing, independent of conscious appetite ratings, and contribute to a deeper understanding of how taste influences neural mechanisms of food perception.

## Figures and Tables

**Table 1:** Group characteristics (means and standard deviations).

	Monosodium Glutamate M (SD)	Water M (SD)
**Age (years)**	24.93 (8.42)	25.98 (7.92)
**Body mass index**	22.36 (2.89)	23.85 (4.06)
**Hunger level (pre)**	51.20 (27.44)	46.34 (25.36)
**Hunger level (post)**	61.47 (28.35)	55.06 (33.32)
**Valence (pre)**	77.50 (14.65)	76.39 (20.26)
**Valence (post)**	71.32 (19.31)	71.17 (20.07)
**Arousal (pre)**	27.33 (22.25)	30.02 (24.50)
**Arousal (post)**	18.55 (18.34)	22.58 (21.83)
**Fluid ratings**		
**Pleasantness**	29.44 (16.95)	72.43 (20.62)
**Intensity**	55.18 (23.08)	13.02 (16.40)
**Familiarity**	32.03 (22.27)	86.03 (21.25)
**Image ratings (wanting)**		
**Sweets**	41.85 (19.98)	41.12 (23.70)
**Meat**	31.37 (25.39)	31.36 (24.43)
**Fruits**	63.03 (15.57)	65.94 (18.93)
**Vegetables**	50.79 (20.93)	45.35 (19.56)
**Image ratings (liking)**		
**Sweets**	64.39 (16.54)	63.82 (20.12)
**Meat**	47.34 (27.14)	51.00 (23.30)
**Fruits**	79.07 (11.85)	81.33 (11.96)
**Vegetables**	73.31 (16.38)	69.87 (14.67)

**Table 2:** Maxima of estimated cortical activity in the time frames P300 and LPP.

*TF* (*ms*)	*Solution Point*	*Coordinates* (*Talairach, mm*)	*Label*
P300	RAI202	(25, 3, −34)	Right fusiform gyrus
LPP	LAI236 RAS493 LPI425	(−34, 1, −34) (3, 58, 5) (−21, −100, 2)	Left fusiform gyrus Right medial transversal frontopolar gyrus Left occipital gyrus

Footnote: LAI: left anterior inferior; RAS: right anterior superior; RAI: right anterior inferior.

## Data Availability

The original contributions presented in the study are included in the article, further inquiries can be directed to the corresponding author.
